# Characterization of the complete mitochondrial genome of *Spirobolus grahami* (Diplopoda: Spirobolidae) with phylogenetic analysis

**DOI:** 10.1038/s41598-024-57421-3

**Published:** 2024-03-30

**Authors:** Wenwen Zhang, Tianyi Gan, Tangjun Xu, Peng Wang, Jingzhe Tai, Fangzhou Ma

**Affiliations:** 1 Research Center for Biodiversity Conservation and Biosafety/State Environmental Protection Scientific Observation and Research Station for Ecological Environment of Wuyi Mountains/Biodiversity Comprehensive Observation Station for Wuyi Mountains/State Environmental Protection Key Laboratory on Biosafety, Nanjing Institute of Environmental Sciences, Ministry of Ecology and Environment of China, Nanjing, 210042 China; 2 College of Plant Health and Medicine, Qingdao Agricultural University, Qingdao, 266109 China; 3 College of Life Science, Nanjing Forestry University, Nanjing, 210037 China

**Keywords:** Mitochondrial genome, Diplopoda, *Spirobolus grahami*, Phylogenetic analysis, Genetics, Molecular biology, Zoology

## Abstract

Diplopoda is one of the most diverse and important groups of soil arthropods, but little research has been done on their phylogenetic relationship and evolution. Here, we sequenced and annotated the complete mitochondrial genomes of *Spirobolus grahami*. The total mitogenome of *S. grahami* was typical circular, double-stranded molecules, with 14,875 bp in length, including 13 protein-coding genes, 22 tRNAs, two rRNAs, and one control region. Base composition analysis suggested that the mitochondrial sequences were biased toward A and T, with A + T content of 58.68%. The mitogenomes of *S. grahami* exhibited negative AT and positive GC skews. Most of the 13 PCGs had ATN as the start codon, except COX1 start with CGA, and most PCGs ended with the T stop codon. The dN/dS values for most PCGs were lower than 1, suggesting that purifying selection was likely the main driver of mitochondrial PCG evolution. Phylogenetic analyses based on 13 PCGs using BI and ML methods support the classification of genus *Spirobolus* and *Tropostreptus*. *Glomeridesmus spelaeus* is distantly related to the other Diplopoda species.

## Introduction

Invertebrate mitochondrial genome (mitogenome) is typically double-stranded and closed circular molecules, approximately 15–18 kb in length^1,2^. Invertebrate mitogenome consists of 13 protein-coding genes (PCGs), 22 transfer RNAs (tRNAs), 2 ribosomal RNAs (rRNAs), and one non-coding control region (CR)^2,3^. The mitogenome has characteristics such as small size, simple structure and fast evolution, it has been extensively studied and widely used for species identification and molecular phylogeny researches^4,5^.

Diplopoda (millipedes) is one of the most diverse groups of arthropods, with more than 7000 species described^6^. Millipedes *Spirobolus grahami* belongs to the Spirobolidae family of the Diplopoda class^7^. Millipedes are an important part of modern terrestrial ecosystems and play an important role in the decomposition of organic matter^6,8,9^. However, few studies have documented the phylogeny, evolution, behavior, physiology, and ecology of Millipedes^8,10,11^. Therefore, the use of mitogenome might be expected to provide valuable data on their phylogenetic relationship.

In order to further investigate the relationship between the Diplopoda, in this study, we firstly sequenced and characterized the mitogenome of *S. grahami*. The structural organization, nucleotide composition, codon usage, and AT/GC-skew were analyzed. Additionally, we conducted phylogenetic analyses based on 13 PCGs available elsewhere for the purpose of investigating the phylogenetic position of *S. grahami* within Diplopoda, which we believe might be helpful for further evolutionary and phylogenetic studies on millipedes within the Diplopoda.

## Materials and methods

### Sample collection and DNA extraction

Sample used in this study collected from Guilin Seven Star Park (Guilin, China). The collected sample was morphologically characterized based on the images and morphological features on GBIF (https://www.gbif.org/) and MilliBase (https://millibase.org/), with specific reference to Keeton^12^. The collection of the specimen was reviewed and approved by Nanjing Forestry University. Specimen for this study was collected in accordance with Chinese laws. Sample was stored at the Zoology Laboratory of Nanjing Forestry University. Total DNA was extracted from muscular tissue using a FastPure Cell/Tissue DNA Isolation Mini Kit (Vazyme™, Nanjing, China). The remaining tissue was stored at − 20 °C in 90% ethanol to preserve the specimens.

### Next-generation sequencing

Library construction and sequencing were carried out by Novogene (Nanjing, China) on the HiSeq 2500 platform (Illumina Inc., San Diego, USA) following the manufacture’s protocol for 150-bp paired-end reads. Clean reads were used to assemble the full mitogenome in Geneious Prime 2020 using *Spirobolus bungii* (NC056899.1) as the template, and both ends of the final assembly were manually examined for overlap to build a circular mitogenome.

### Annotation and sequence analysis

The BLAST CD-search (https://www.ncbi.nlm.nih.gov/Structure/cdd/wrpsb.cgi) and MITOS Webserver (http://mitos.bioinf.uni-leipzig.de/index.py) were used to detect PCGs, tRNAs, rRNAs, and CR^13–15^. The gene map of the mitogenome was generated with the CG view Server (http://cgview.ca/)^16^. Nucleotide compositional differences between genes were calculated according to the following formulae: AT-skew = (A − T)/(A + T) and GC-skew = (G − C)/(G + C)^17,18^. Relative synonymous codon usage (RSCU) was calculated in MEGA X and image rendered by PhyloSuite v1.2.1^19,20^. The synonymous replacement rate (dS), non-synonymous replacement rate (dN), and the ratio of non-synonymous replacement rate to synonymous replacement rate (dN/dS) were determined using MEGA X for *Spirobolus* species^19^.

### Phylogenetic analysis

We constructed a concatenated set of base sequences of the 13 PCGs from 24 species to study the phylogenetic relationship in Diplopoda (Table 1). *Lithobius forficatus* was used as an outgroup. Phylogenetic analyses were conducted for each dataset using Bayesian inference (BI) and maximum likelihood (ML) methods. All operations were performed in PhyloSuite v1.2.1^20^. MAFFT was used to perform multiple sequence alignment, with strategy of L-INS-i. ModelFinder was used to select the best-fit model. The best fit models of BI were GTR + F + I + G4 for COX1, COX2, COX3, ND1, ND2, ND4, ND4L, ND5, ND6, HKY + F + I + G4 for ATP6, ND3, Cytb, and HKY + F + G4 for ATP8. The best fit models of ML were GTR + F + R4 for COX1, COX2; GTR + F + R5 for ND1, ND4, ND4L, ND5, HKY + F + I + G4 for ATP6, HKY + F + G4 for ATP8, TIM2 + F + I + G4 for COX3, TPM3u + F + I + G4: Cytb, ND3, TPM3u + F + I + I + R3 for ND2, ND6. BI tree was performed with MrBayes 3.2.6 and run for 1,000,000 generations, with a burn-in of 25% trees, while ML tree was performed in the IQ-TREE^21,22^. The phylogenetic trees were viewed and edited using iTOL (https://itol.embl.de/)^23^.

**Table 1:** The mitogenomes used in phylogenetic analyses.

Species	Accession no	Size (bp)
*Abacion magnum*	JX437062.1	15,160
*Anaulaciulus koreanus*	KX096886.1	14,916
*Appalachioria falcifera*	JX437063.1	15,282
*Archispirostreptus gigas*	MT394525.1	15,177
*Asiomorpha coarctata*	KU721885.1	15,644
*Brachycybe lecontii*	JX437064.1	15,115
*Chaleponcus netus*	MT394513.1	15,093
*Epanerchodus koreanus*	MT898420.1	15,581
*Glomeridesmus spelaeus*	MG372113.1	14,819
*Macrolenostreptus orestes*	MT394512.1	15,367
*Narceus annularus*	AY055727.1	14,868
*Prionopetalum kraepelini*	MT394524.1	15,114
*Pseudotibiozus cerasopus*	MT394506.1	15,121
*Spirobolus bungii*	MT767838.1	14,879
*Spirobolus grahami*	OR038162	14,875
*Spirobolus walkeri*	OR078377.1	14,879
*Tropostreptus austerus*	MT394523.1	15,261
*Tropostreptus droides*	MT394522.1	15,172
*Tropostreptus hamatus*	MT394508.1	15,156
*Tropostreptus kipunji*	MT394503.1	15,170
*Tropostreptus microcephalus*	MT394516.1	15,169
*Tropostreptus severus*	MT394517.1	15,209
*Tropostreptus sigmatospinus*	MT394504.1	15,176
*Lithobius forficatus*	AF309492.1	15,695

## Results and discussion

### Mitochondrial genome organization

**Figure 1:**
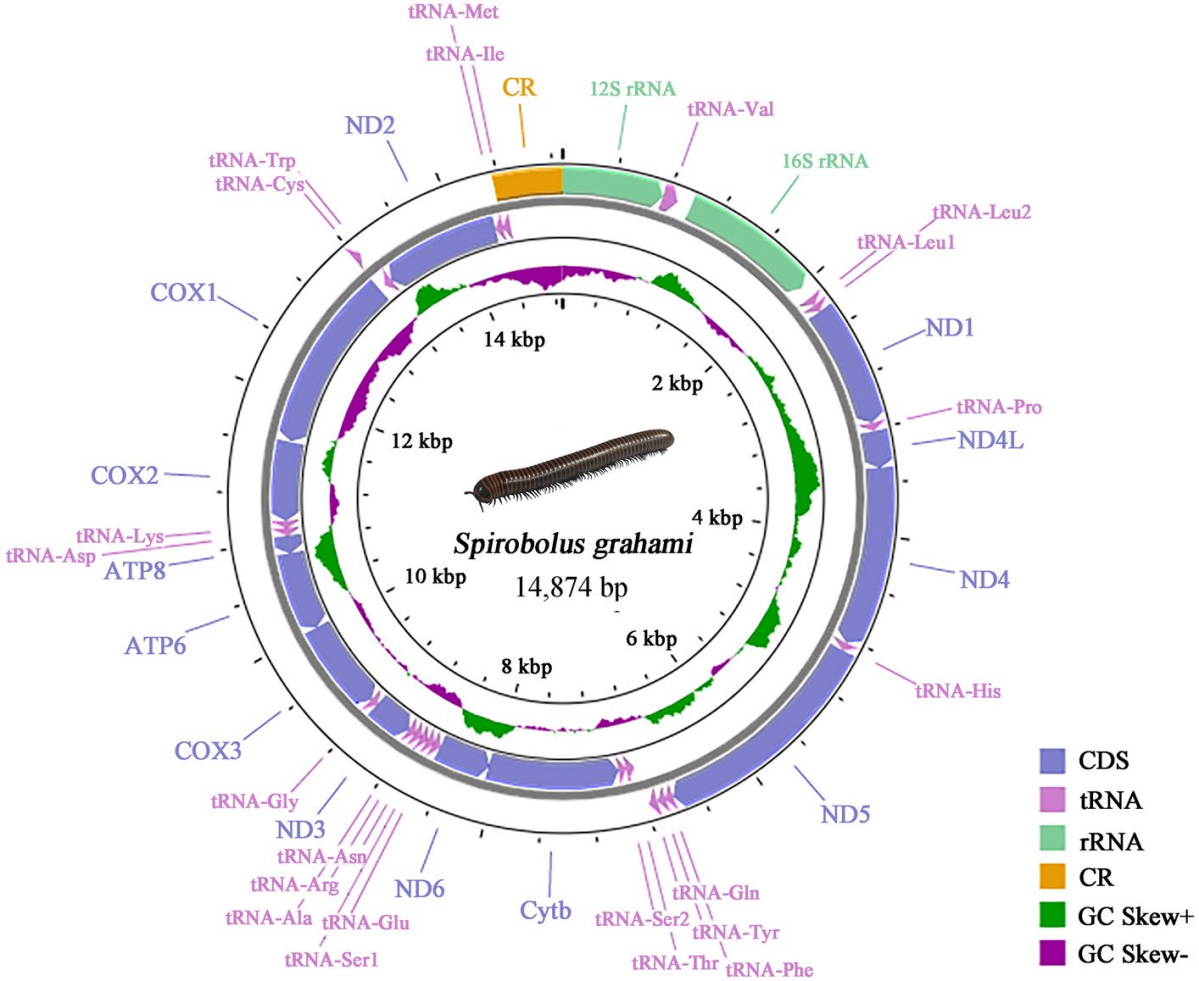
Mitochondrial genome of *S. grahami*. Yellow blocks: CR; green blocks: rRNAs; light purple blocks: tRNAs; dark purple blocks: PCGs.

The total mitogenome of *S. grahami* was typical circular, double-stranded molecules, with 14,875 bp in length (Fig. 1). The mitogenome has been submitted to GenBank (Table 1). Mitogenomes of *S. grahami* encoded all 37 classical mitochondrial genes (13 PCGs, 22 tRNAs, and 2 rRNAs) and one CR. In this mitogenome, 15 genes (four PCGs, two rRNAs, and nine tRNAs) were transcribed from the majority strand (J strand), and the remaining 22 genes were transcribed from the minority strand (N strand) (Table 2), which is identical to *S. bungii* of the same genus^11^. The gene order of *S. grahami* was also consistent with that of *S. bungii and Spirobolus walkeri* in the same genus^11^. The gene order of millipede mitogenome is diverse^24^, but the gene order of this genus is relatively stable.

**Table 2:** Annotation and organization of the complete mitogenome of *S. grahami*.

Gene	Position		Size (bp)	Orientation	Codon		Intergenic nucleotides
	From	To			Start	Stop	
12S rRNA	1	757	757	+			
tRNA-Val	757	875	119	+			0
16S rRNA	979	2011	1033	+			104
tRNA-Leu1	2103	2165	63	+			92
tRNA-Leu2	2166	2228	63	+			1
ND1	2229	3150	922	+	ATA	T	1
tRNA-Pro	3151	3213	63	+			1
ND4L	3215	3496	282	+	ATG	TAG	2
ND4	3490	4834	1345	+	ATG	T	− 6
tRNA-His	4835	4897	63	+			1
ND5	4898	6599	1702	+	ATT	T	1
tRNA-Phe	6600	6660	61	+			1
tRNA-Tyr	6657	6718	62	+			− 3
tRNA-Gln	6721	6788	68	+			3
tRNA-Thr	6829	6890	62	–			41
tRNA-Ser2	6895	6958	64	–			5
Cytb	6959	8075	1117	–	ATG	T	1
ND6	8068	8523	456	–	ATT	TAA	− 7
tRNA-Glu	8524	8584	61	–			1
tRNA-Ser1	8585	8641	57	–			1
tRNA-Asn	8642	8704	63	–			1
tRNA-Arg	8704	8765	62	–			0
tRNA-Ala	8765	8826	62	–			0
ND3	8827	9172	346	–	ATA	T	1
tRNA-Gly	9173	9235	63	–			1
COX3	9236	10,013	778	–	ATG	T	1
ATP6	10,014	10,689	676	–	ATG	T	1
ATP8	10,683	10,838	156	–	ATT	TAA	− 6
tRNA-Asp	10,839	10,900	62	–			1
tRNA-Lys	10,900	10,965	66	–			0
COX2	10,966	11,643	678	–	ATG	TAA	1
COX1	11,647	13,176	1530	–	CGA	TAA	4
tRNA-Cys	13,182	13,244	63	+			6
tRNA-Trp	13,237	13,298	62	–			− 7
ND2	13,299	14,298	1000	–	ATA	T	1
tRNA-Met	14,299	14,361	63	–			1
tRNA-Ile	14,362	14,425	64	–			1
CR	14,426	14,875	450				1

Base composition analysis suggested that this mitogenome was biased toward A and T, the content ratio of A + T is 58.68% (Table 3), which is consistent with a previous study^11^. Besides, the PCGs, tRNAs, rRNAs, and CR were all biased in nucleotide composition (A + T > G + C), which is consistent with other invertebrate researches^25,26^. The AT-skew of *S. grahami* was negative, while the GC-skew was positive. The low GC-skew values of the analyzed mitogenome indicated the occurrence of more Cs than Gs. However, the AT-skew of tRNAs and CR were slight positive.

**Table 3:** Nucleotide composition and skewness of *S. grahami* mitogenome.

	Total genome	PCGs	tRNAs	rRNAs	CR
Size (bp)	14,875	10,988	1376	1790	450
A (%)	26.01	24.37	34.16	29.16	38.22
T (%)	32.67	32.07	30.60	34.58	33.56
G (%)	29.23	31.40	18.68	24.64	16.22
C (%)	12.09	12.16	16.57	11.62	12.00
A + T (%)	58.68	56.44	64.76	63.74	71.78
G + C (%)	41.32	43.56	35.25	36.26	28.22
AT-skew	− 0.1135	− 0.1364	0.0550	− 0.0850	0.0649
GC-skew	0.4148	0.4417	0.0599	0.3591	0.1495

Multiple overlaps between contiguous genes were calculated. There were five gene overlaps in this mitogenome, ranging from 3 to 7 bp. The longest overlap region of the mitogenome was found between Cytb and ND6, as well tRNA-Cys and tRNA-Trp, with 7 bp in length.

### Protein-coding genes and codon usage

The total length of the PCGs was 10,988 bp, accounting for 73.87%. Four PCGs, ND1, ND4L, ND4, and ND5 were transcribed from the J-stand, and the other PCGs from the N-strand. The sizes of 13 PCGs ranged from 156 (ATP8) to 1702 bp (ND5) in the mitogenome. The start codon of all PCGs is ATN (ATG, ATT, and ATA), except COX1 starts with the CTA codon. This unusual start codon, CTA, have previously been reported in *Spirobolus*^11^. In addition, three stop codons were found in the PCGs of *S. grahami*, namely TAA, TAG, and T. In the mitogenome, the occurrence frequency of the stop codon T was higher than those of the other two stop codons, while the stop codon TAG occurred the least.

**Figure 2:**
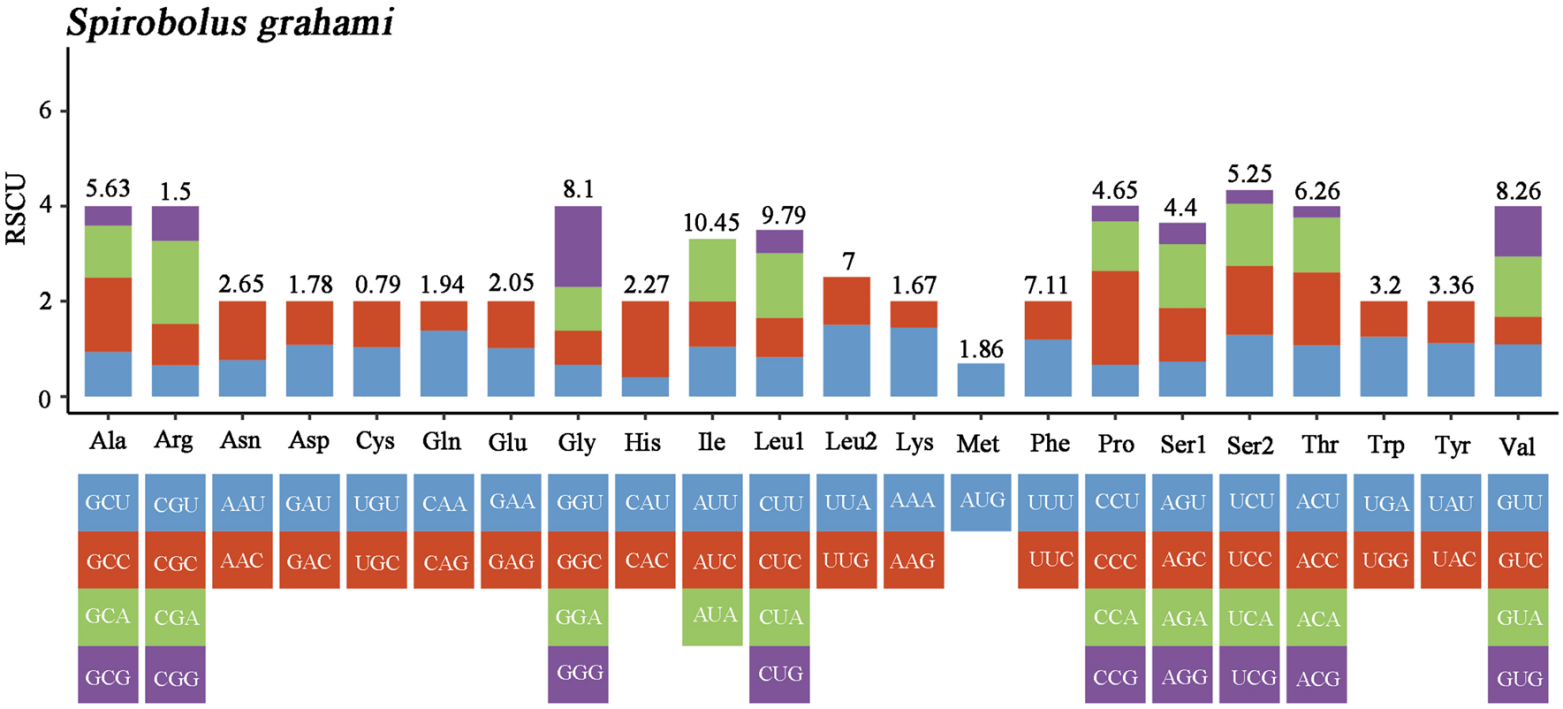
Relative synonymous codon usage of *S. grahami*, the stop codon is not included.

The RSCUs of the PCGs in the mitogenome were calculated, as shown in Fig. 2. The RSCUs analysis of *S. grahami* showed that codons tended to use more A or T at the third codon, which is consistent with some previous studies^27,28^. The dN/dS of the PCGs in the mitogenome of *Spirobolus* (*S bungii*, *S. grahami*, and *S. walkeri*) were calculated, too (Table 4). In evolutionary analysis, it is necessary to understand the rate at which dN and dS mutations occur, analyzing their ratios to detect selective pressures, if any, among PCGs. In this study, ND4L having the lowest evolutionary rate, and COX1 having the highest sequence variability. The faster evolution of COX1 of the genus *Spirobolus* might result in greater amino acid diversity, indicating its potential as an effective marker for classification. The dN/dS values for most PCGs were lower than 1, suggesting that purifying selection was likely the main driver of mitochondrial PCG evolution^29^.

**Table 4:** The dN/dS values among *Spirobolus* species.

PCGs	dN/dS
ND1	0.0857
ND4L	0.0645
ND4	0.8191
ND5	0.1034
Cytb	0.9521
ND6	0.1418
ND3	0.1258
COX3	3.2000
ATP6	2.2500
ATP8	0.6857
COX2	2.0000
COX1	6.5000
ND2	1.2609

### Transfer RNA, ribosomal RNA genes and control regions

**Figure 3:**
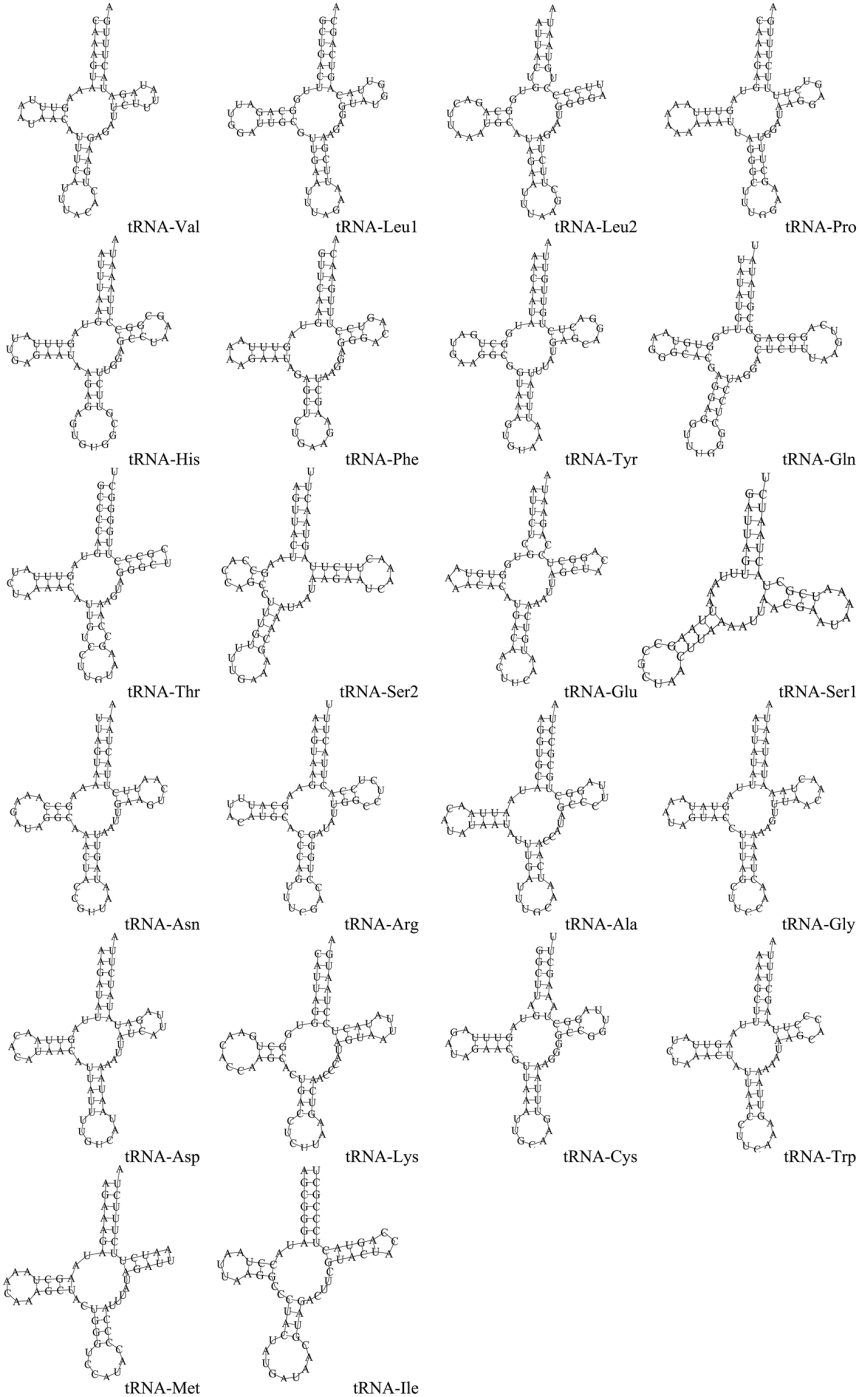
Secondary structure of 22 tRNA genes from the *S. grahami* mitogenome.

22 tRNAs and two rRNAs were discontinuously distributed throughout the whole mitogenome. The tRNA genes of the mitogenome were 1376 bp, which account for 9.3% of the entire mitogenome. There were nine tRNAs from the J-strand and 14 transcribed from the N-strand. Among all secondary structures of the 22 tRNA genes from the *S. grahami* mitogenome, except for tRNA-Ser1, all had a typical cloverleaf structure (Fig. 3), as observed in other Diplopoda mitogenomes^8,11,30^. The 16S rRNA (1033 bp) was encoded between tRNA-Val and tRNA-Leu1, and the 12S rRNA was 757 bp long. The total size of the two rRNAs was 1790 bp, accounting for 12.03%.

One CR was found between the genes tRNA-Ile and 12S rRNA in the mitogenome, with 450 bp in length, accounting for 3.03%. The content of A + T is 71.78%, consistent with research that mitochondrial CR is typically characterized by high A + T content in most invertebrates^25,31,32^.

### Phylogenetic analyses

**Figure 4:**
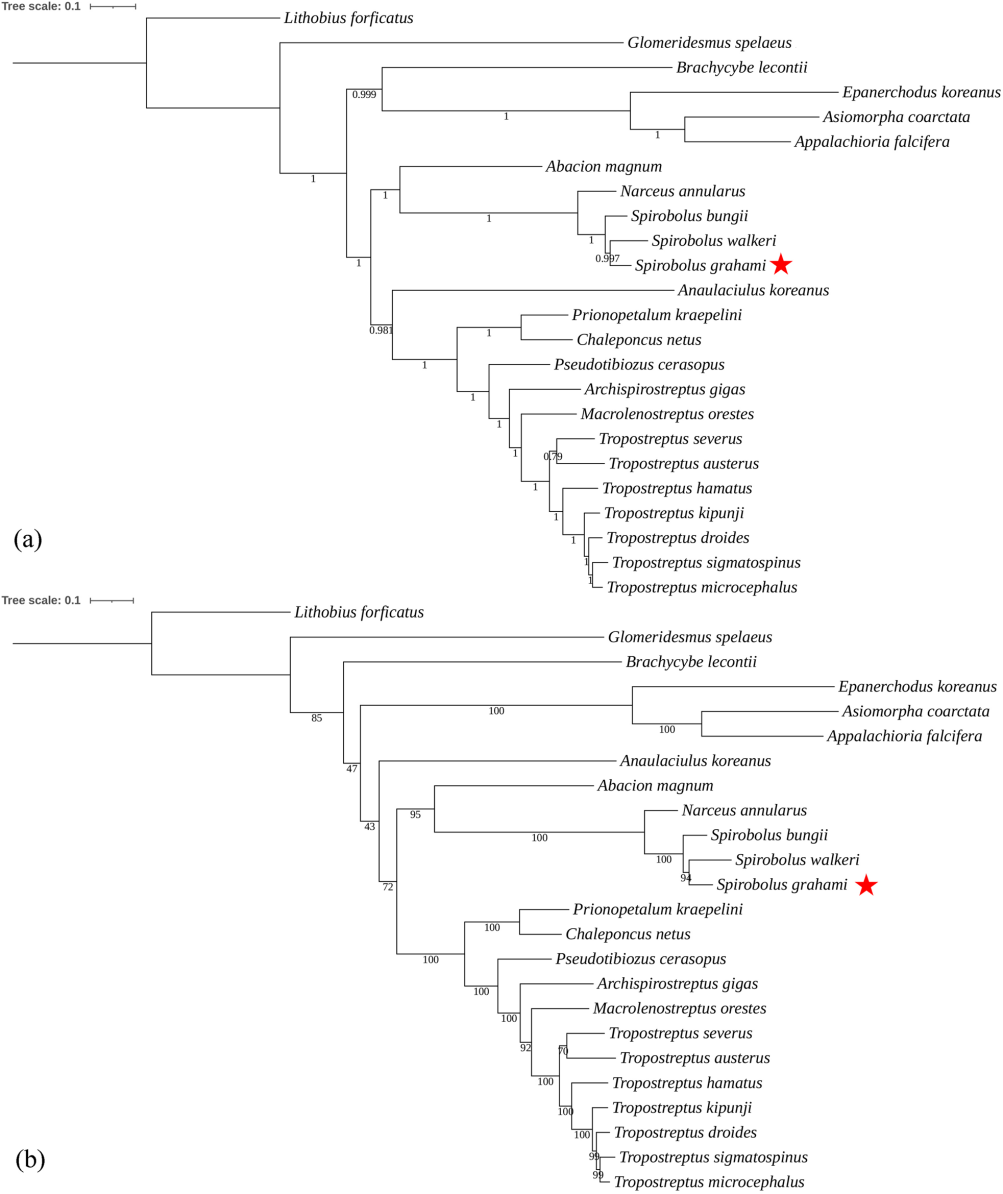
Phylogenetic trees of 23 Diplopoda species and an outgroup (*Lithobius forficatus*) based on 13 PCGs using the BI (**a**) and ML (**b**) method.

We included 23 species of Diplopoda in the phylogenetic analyses and selected *L. forficatus* in Chilopoda as an outgroup to root the phylogenetic trees, using BI and ML methods. Phylogenetic trees were constructed based on sequences of 13 PCGs (Fig. 4). The topologies of the BI and ML trees were similar to each other. *S. grahami* is clustered together with *S. bungii* and *S. walkeri*, which belong to the same genus *Spirobolus*. *Narceus annularus* is closely related to genus *Spirobolus*, which is consistent with the result of previous study^11^. *Glomeridesmus spelaeus* is distantly related to the other Diplopoda species, similar to the previous study^33^. In addition, phylogenetic trees also support the classification of genus *Tropostreptus*. Previous study on millipede mitochondria have shown that genus *Tropostreptus* is phylogenetically more closely related to *Archispirostreptus gigas* and *Macrolenostreptus orestes*^34^. The results of the our study on the phylogenetic analysis of mitochondria also support this. We demonstrate that the mitogenome might be an effective tool for millipede classification. Our study shows that mitogenome sequences are effective molecular markers for studying the phylogenetic relationships and evolution within Diplopoda, but the data that covered only 22 species, meaning it’s still limited.

## Conclusions

The mitogenome of *S. grahami* was determined to be 14,875 bp in length, with A + T content of 58.68%. The nucleotide composition showed that the mitogenomes of *S. grahami* exhibited negative AT and positive GC skews. The COX1 having the highest sequence variability. The dN/dS values for most PCGs were lower than 1, suggesting that purifying selection was likely the main driver of mitochondrial PCG evolution Both BI and ML trees support the classification of genus *Spirobolus* and *Tropostreptus*. Our results would contribute to the future resolution of phylogenetic relationships in Diplopoda.

## Data Availability

Representative nucleic acid sequences reported in this paper have been submitted to NCBI (https://www.ncbi.nlm.nih.gov/) GenBank database under the accession numbers OR038162.
