# Author Correction: Characterization of the complete mitochondrial genome of *Spirobolus grahami* (Diplopoda: Spirobolidae) with phylogenetic analysis

**DOI:** 10.1038/s41598-024-61707-x

**Published:** 2024-05-13

**Authors:** Wenwen Zhang, Tianyi Gan, Tangjun Xu, Peng Wang, Jingzhe Tai, Fangzhou Ma

**Affiliations:** 1 Research Center for Biodiversity Conservation and Biosafety/State Environmental Protection Scientifc Observation and Research Station for Ecological Environment of Wuyi Mountains/Biodiversity Comprehensive Observation Station for Wuyi Mountains/State Environmental Protection Key Laboratory on Biosafety, Nanjing Institute of Environmental Sciences, Ministry of Ecology and Environment of China, Nanjing, 210042 China; 2 College of Plant Health and Medicine, Qingdao Agricultural University, Qingdao, 266109 China; 3 College of Life Science, Nanjing Forestry University, Nanjing, 210037 China

Correction to: *Scientific Reports* 10.1038/s41598-024-57421-3, published online 30 March 2024

The original version of this Article contained an error in the Funding section.

“This work was supported by the biodiversity investigation, observation and assessment program (2019–2023) of Ministry of Ecology and Environment of China (2110404). National Key R&D Programme of China (Grant No. 2021YFC2600400).”

now reads

“This work was supported by the National Key R&D Programme of China (Grant No. 2021YFC2600400) and the Biodiversity Investigation, Observation and Assessment program (2019–2023) of the Ministry of Ecology and Environment of China (2110404).”

The original Article has been corrected.

